# Achieving Highly Efficient Photocatalytic Hydrogen Evolution through the Construction of g-C_3_N_4_@PdS@Pt Nanocomposites

**DOI:** 10.3390/molecules29020493

**Published:** 2024-01-19

**Authors:** Ligang Ma, Chao Lin, Wenjun Jiang, Shun Yan, Huilin Jiang, Xiang Song, Xiaoqian Ai, Xiaoxiao Cao, Yihuan Ding

**Affiliations:** 1 School of Electronic Engineering, Nanjing Xiaozhuang University, Nanjing 211171, China; maligang186@163.com (L.M.); 21090305@njxzc.edu.cn (C.L.); 200903241@njxzc.edu.cn (W.J.); 2019008@njxzc.edu.cn (S.Y.); huilin.jiang@njxzc.edu.cn (H.J.); songxiang@njxzc.edu.cn (X.S.); yihuanding@njxzc.edu.cn (Y.D.); 2 School of Physics and Information Engineering, Jiangsu Province Engineering Research Center of Basic Education Big Data Application, Jiangsu Second Normal University, Nanjing 210013, China; 200903226@njxzc.edu.cn

**Keywords:** g-C_3_N_4_ nanosheet, PdS, nanocomposites, photocatalytic hydrogen production

## Abstract

Selective supported catalysts have emerged as a promising approach to enhance carrier separation, particularly in the realm of photocatalytic hydrogen production. Herein, a pioneering exploration involves the loading of PdS and Pt catalyst onto g-C_3_N_4_ nanosheets to construct g-C_3_N_4_@PdS@Pt nanocomposites. The photocatalytic activity of nanocomposites was evaluated under visible light and full spectrum irradiation. The results show that g-C_3_N_4_@PdS@Pt nanocomposites exhibit excellent properties. Under visible light irradiation, these nanocomposites exhibit a remarkable production rate of 1289 μmol·g^−1^·h^−1^, marking a staggering 60-fold increase compared to g-C_3_N_4_@Pt (20.9 μmol·g^−1^·h^−1^). Furthermore, when subjected to full spectrum irradiation, the hydrogen production efficiency of g-C_3_N_4_@PdS@Pt-3 nanocomposites reaches an impressive 11,438 μmol·g^−1^·h^−1^, representing an eightfold enhancement compared to g-C_3_N_4_@Pt (1452 μmol·g^−1^·h^−1^) under identical conditions. Detailed investigations into the microstructure and optical properties of g-C_3_N_4_@PdS catalysts were conducted, shedding light on the mechanisms governing photocatalytic hydrogen production. This study offers valuable insights into the potential of these nanocomposites and their pivotal role in advancing photocatalysis.

## 1. Introduction

Hydrogen (H_2_) [1], renowned as the lightest gas globally, boasts a high combustion temperature, yielding water upon combustion [2]. Thus, it stands as a pivotal clean energy source, offering promise in alleviating the dual pressures of energy scarcity and environmental pollution [3]. Diverse methods exist for hydrogen production, encompassing electric water decomposition [4], metal–acid reactions, thermal compound decomposition [5], steam reforming of natural gas [6], and the photocatalytic splitting of water [7,8,9]. While the initial three methods exhibit drawbacks, photocatalytic hydrogen production via water splitting is heralded as a green and sustainable avenue for solar energy conversion [10,11,12]. In this process, the development of a highly efficient photocatalyst remains pivotal for its wider application and industrialization. Semiconductor materials, deemed ideal photocatalysts, necessitate strong light absorption capabilities, an appropriate band structure, abundant reactive sites, and efficient carrier separation [13].

Numerous semiconductors, including metal–organic frameworks [14], metal oxide (TiO_2_ [15], ZnO [16], CeO_2_ [17], CuO [18], etc.), carbon-related compounds (GO [19], C_3_N_4_ [20], etc.), and various metal sulfides (CdS [21,22], ZnIn_2_S_4_ [23], CuS [24], etc.), serve as widely employed photocatalysts for hydrogen production. Within this realm, the graphene-like two-dimensional structure of C_3_N_4_ (g-C_3_N_4_) stands out as a typical polymer semiconductor [25]. The C-N atoms in its structure exhibit sp^2^ hybridization, forming a highly delocalized π-conjugated system akin to graphene’s layered structure [26]. Noteworthy for its excellent chemical and thermal stability, visible light absorption, non-toxicity, mineral abundance, and simple preparation process, g-C_3_N_4_ has garnered increasing attention in recent years across multiple domains, emerging as a focal point of research [27,28]. However, inherent photogenerated carrier recombination limits the overall photocatalytic activity of pure g-C_3_N_4_, posing a challenge for substantially enhancing its efficiency. Consequently, various strategies have been explored to improve g-C_3_N_4′_s photocatalytic efficiency, including modifications [29,30,31,32] and heterojunction construction with other semiconductors [33,34,35].

Moreover, the deposition of co-catalysts on nanomaterial surfaces has proven pivotal in facilitating charge separation by swiftly capturing electrons or holes, consequently elevating photocatalytic performance. A gamut of precious metals (such as Pt [36], Pd [37], Rh [38]), metal oxides (NiO [39], CuO [40]), and metal sulfides (MoS_2_ [41]) have found application as co-catalysts, augmenting photocatalytic hydrogen evolution. Notably, previous studies have highlighted PdS as a promising co-catalyst in composite structures with dual co-catalysts, as seen in Pt-PdS/Cd_0.5_Zn_0.5_S [42], PANI@CdS@PdS [43], and Pd@CdS@PdS [44]. The incorporation of PdS enhances the ability for charge separation and promotes excellent photo-stability. Moreover, the introduction of PdS as a co-catalyst reduces the activation energy and fosters surface oxidation-reduction reactions [45]. Leveraging these advantageous characteristics of PdS, we opted to utilize it as a co-catalyst to enhance the photocatalytic production of H_2_ in conjunction with g-C_3_N_4_. This strategy of employing PdS as a catalyst to enhance the photocatalytic activity of g-C_3_N_4_ for H_2_ production has not been previously reported.

Thus, this study focuses on synthesizing g-C_3_N_4_ nanosheets via secondary pyrolysis of melamine, introducing PdS as a hole-trapping agent. The concentration of PdS in g-C_3_N_4_ nanosheets was regulated by controlling the Pd and S sources. Comprehensive investigations into the microstructure, morphology, optical properties, and valence states of g-C_3_N_4_ under varied PdS concentrations were conducted. Subsequently, the photocatalytic hydrogen production performance of the g-C_3_N_4_ nanocomposites under Pt and PdS co-catalysis was evaluated under visible light and full-spectrum irradiation. Detailed scrutiny of the photocatalytic mechanism and analysis thereof revealed significant enhancement in the photocatalytic hydrogen production performance of g-C_3_N_4_@PdS@Pt nanocomposites at an optimal PdS concentration.

## 2. Results and Discussion

**Figure 1:**
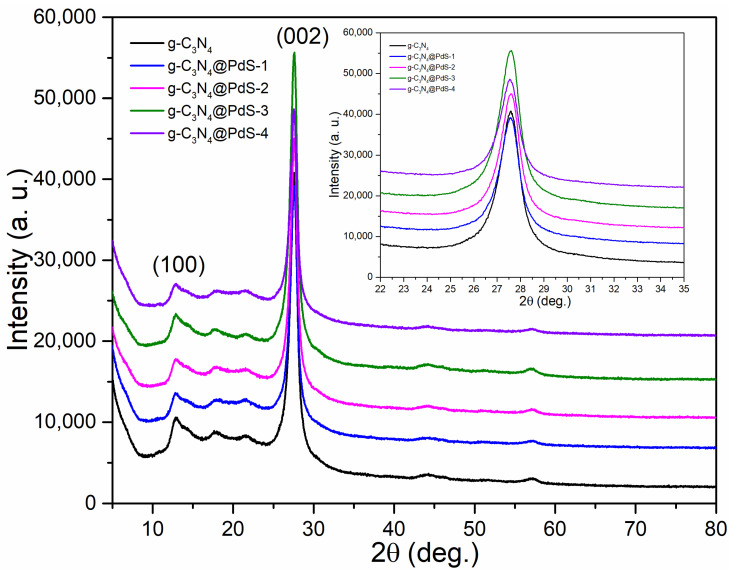
XRD pattern of g-C_3_N_4_ and g-C_3_N_4_@PdS nanocomposites. The illustration is a magnification of the (002) diffraction peak.

Figure 1 displays the X-ray diffraction patterns (XRD) of both g-C_3_N_4_ and g- C_3_N_4_@PdS nanocomposites with adjustable PdS content. Notably, two prominent reflections appear at 2θ = 27.45, and 12.8°. The reflection at =27.5° aligns with the (002) diffraction peak of hexagonal phase in JPCDS 87-1526 [46], signifying the distinct interlayer stacking of the conjugated aromatic groups. Conversely, the faint reflection at =12.8° corresponds to the (100) plane, indicative of the in-plane structure of tri-s-triazine units. Comparatively, no new diffraction peaks attributable to PdS emerge in the g-C_3_N_4_@PdS nanocomposites. This is mainly due to the low PdS content, and may also be due to the high dispersion of PdS on the surface of g-C_3_N_4_ nanosheets, as reported by reference [47,48]. However, there is a noticeable reduction in the intensities and broadening of the (002) and (100) peaks, evident in the magnified illustration within Figure 1. This diminished intensity suggests a disruption in the interlayer structure, potentially attributed to several factors. The first reason is the ultrasonic stripping process undergone by a g-C_3_N_4_ during nanocomposite preparation. Additionally, the insertion of PdS nanoparticles between g-C_3_N_4_ layers contributes to this alteration. Moreover, as the quantity of PdS increases, the (002) diffraction peak shifts towards a lower angle, providing further evidence of successful PdS insertion between the g-C_3_N_4_ lamellas.

**Figure 2:**
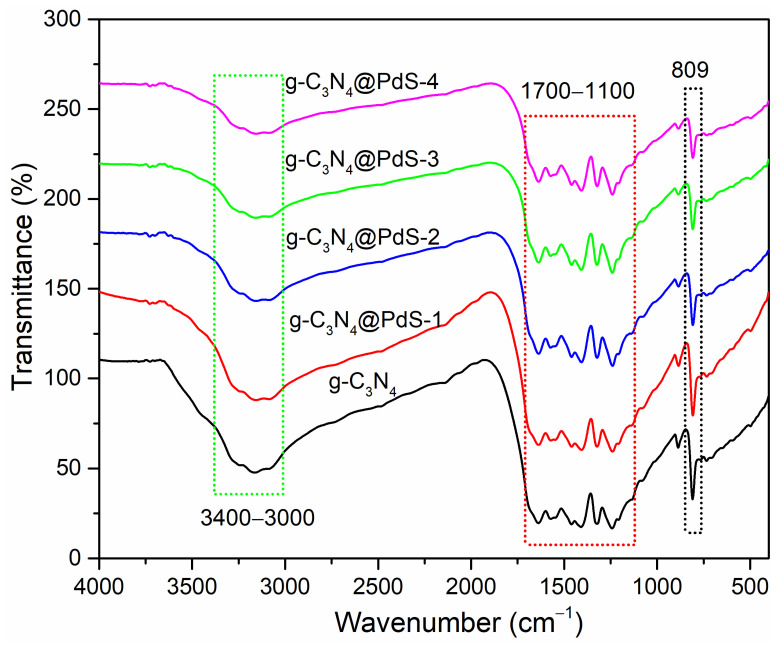
FTIR transmission spectra of g-C_3_N_4_ and g-C_3_N_4_ varying the PdS concentration.

Figure 2 shows the Fourier Transform Infrared spectroscopy (FTIR) of sample g-C_3_N_4_ and g-C_3_N_4_@PdS nanocomposites. It is apparent that the characteristic peaks of the g-C_3_N_4_@PdS nanocomposites remain relatively consistent when compared to the pure g-C_3_N_4_. The characteristic stretching peaks were in three main regions: 809, 1100–1700, and 3000–3400 cm^−1^. Specifically, the wide vibration band at 3000–3400 cm^−1^ signifies the stretching vibration peak associated with N-H, residual amino groups and O-H adsorbed on the surface of g-C_3_N_4_ [49,50]. And the multiple strong vibration bands within the range of 1100–1700 cm^−1^ arise from the unique stretching vibration peak related to the C-N heterocyclic ring [51]. Additionally, the peak at 809 cm^−1^ aligns with the characteristic vibration of the triazine units [52,53]. Furthermore, the figure illustrates that, as the amount of PdS increases, the vibration mode within the 809 and 3000–3400 cm^−1^ regions weakens, suggesting successful insertion of PdS into the g-C_3_N_4_ layer. To delve deeper into the microstructure analysis of g-C_3_N_4_@PdS catalysts, we conducted TEM characterization.

**Figure 3:**
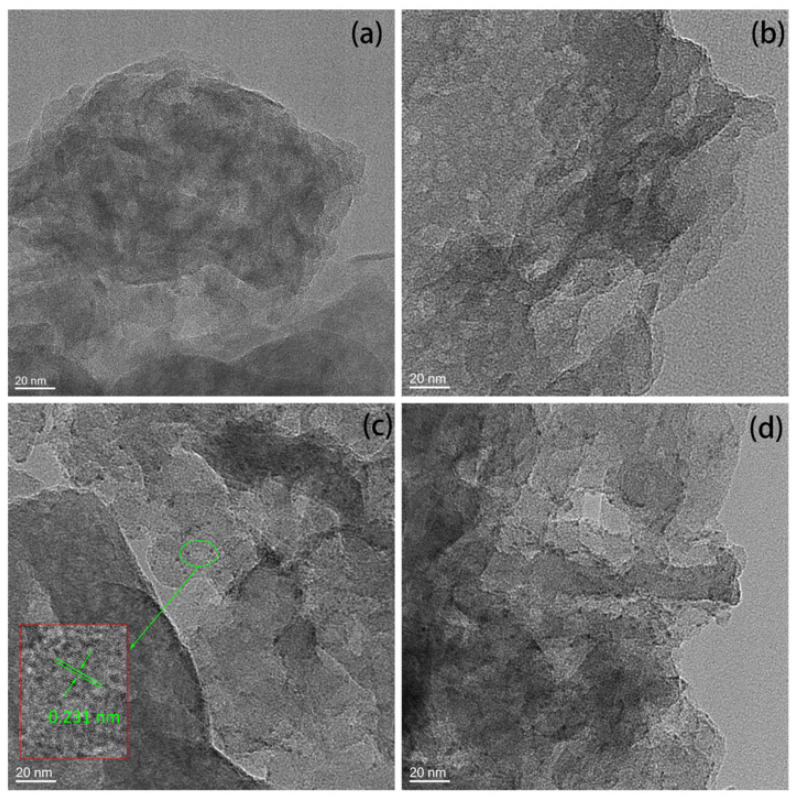
TEM morphology of (**a**) g-C_3_N_4_, (**b**) g-C_3_N_4_@PdS-1, (**c**) g-C_3_N_4_@PdS-3 and (**d**) g-C_3_N_4_@PdS-4 nanocomposites. The inset in (**c**) is the corresponding high-magnification TEM morphology.

Figure 3 illustrates the transmission electron microscopy (TEM) of both g-C_3_N_4_ and g-C_3_N_4_@PdS nanocomposites. The g-C_3_N_4_ exhibits a uniform composition with consistent thickness. Upon the introduction of Pd and S sources, black nanoparticles attached to the nanosheet layer (Figure 3b). Moreover, as the quantity of Pd and S sources increases, there is a proportional rise in the number of nanoparticles, notably evident in g-C_3_N_4_@PdS-3. The distribution of these black nanoparticles appears uniform. The size of the nanoparticles is about 5 nm. Detailed examination via high-resolution TEM (HRTEM, depicted in Figure 3c) reveals a lattice fringe measuring 0.231 nm, corresponding to the (202) crystal plane of the PdS. This observation confirms that the attached nanoparticles consist of PdS. Furthermore, an escalation in the Pd and S sources leads to a higher loading capacity of PdS nanoparticles.

**Figure 4:**
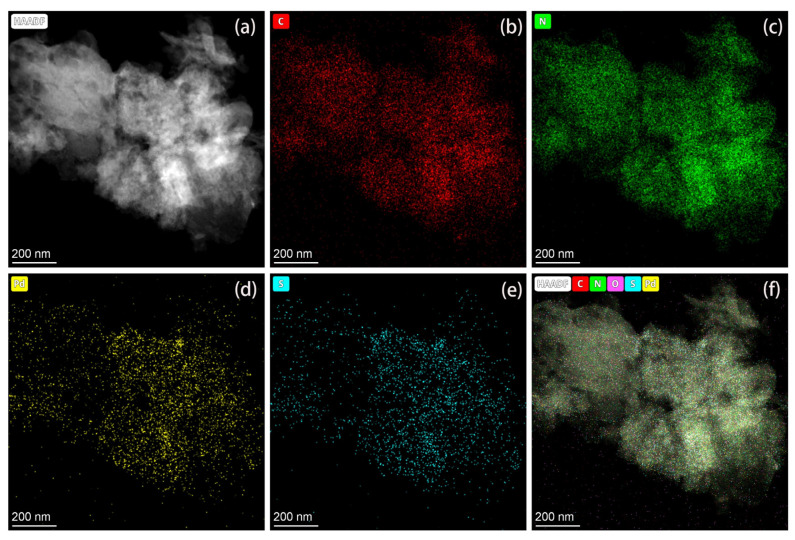
Element mapping of g-C_3_N_4_@PdS-3 nanocomposites: (**a**) high-angle annular dark-field STEM image, (**b**) C element, (**c**) N element, (**d**) Pd element, (**e**) S element, and (**f**) overlay diagram of element mapping results.

Figure 4 illustrates the characterization of the typical g-C_3_N_4_@PdS-3 nanocomposites through high-angle annular dark-field STEM imaging and energy dispersive spectroscopy (EDS) elemental mappings. In the high-angle annular dark-field image, the brightness levels correspond to the distribution of Pd and S in nanoparticles, and, conversely, reveal the distribution of C and N. Analysis of each element’s mapping shows a uniform distribution of C, N, Pd, and S elements within the g-C_3_N_4_@PdS-3 nanocomposites. Given the low PdS dosage, the density of Pd and S on the nanosheet is relatively low.

**Figure 5:**
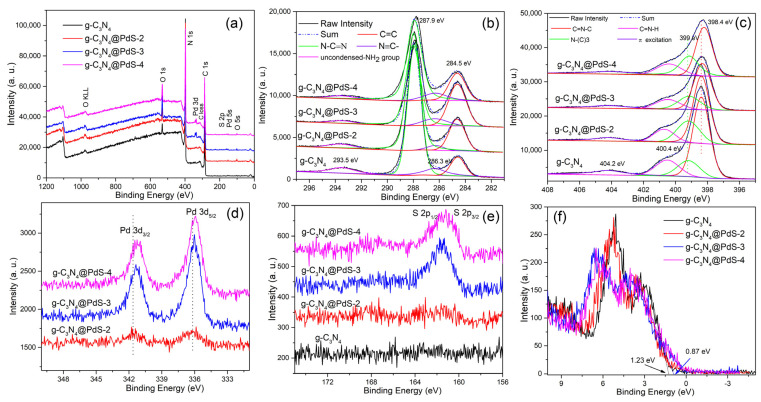
XPS spectra of g-C_3_N_4_, g-C_3_N_4_@PdS-2, g-C_3_N_4_@PdS-3, and g-C_3_N_4_@PdS-4: (**a**) survey spectra, (**b**) C1s, (**c**) N 1s, (**d**) Pd 3d, (**e**) S 2p, and (**f**) valence band.

To analyze the chemical bond state and molecular structure on the material surface, both g-C_3_N_4_ and the g-C_3_N_4_@PdS nanocomposites underwent characterization via X-ray photoelectron spectra (XPS). All XPS spectra were calibrated by aligning the C=C binding energy position to 284.5 eV. Figure 5a illustrates the elemental composition of g-C_3_N_4_, revealing the presence of C, N, and O elements exclusively. As the quantity of Pd and S sources increases, a gradual emergence of weak binding energy associated with Pd and S elements is observed. Simultaneously, intensity of the Pd and S binding energy peaks stability intensifies. This phenomenon indicates the successful recombination of Pd and S within the g-C_3_N_4_ layer, signifying a progressive increase in PdS content. The C1s spectra were analyzed in Figure 5b, revealing four distinctive peaks through Gaussian fitting. These peaks, situated at approximately ~284.5, 286.3, 287.9, and 293.5 eV, correspond to graphitic-like carbon species (C=C or C–C), the N≡C-bond between the sp^2^ C atom and NH groups in the aromatic ring, sp^2^ hybridized carbon atoms bonded with N (N–C=N), and carbon attached to uncondensed-NH_2_ groups. Similarly, the N1s spectrum in Figure 5c, segmented via Gaussian fitting, displayed four peaks at 398.4 (C–N=C), 399 (N–(C)_3_), 400.4 (C=N–H), and 404.2 eV (π excitation), respectively. The Pd 3d spectrum showcased peaks at 336.2 and 341.6 eV (Figure 5d), attributed to Pd 3d_5/2_, and Pd 3d_3/2_, indicating the presence of Pd^2+^. Regarding the S 2p spectrum in Figure 5e, division into two peaks with binding energies at 161.1 and 162.3 corresponded to S2p_3/2_ and S2p_1/2_, respectively, suggesting the existence of S^2−^ in the nanocomposites. Comparison with g-C_3_N_4_ revealed a downward shift in the binding energy of C, N, Pd, and S with increasing Pd and S sources. This shift implies an altered internal chemical bond within the nanocomposites post-formation, suggesting an interface interaction between g-C_3_N_4_ and PdS. Furthermore, the XPS valence band spectra provide insights into the valence band position (E_VB, XPS_) of both g-C_3_N_4_ and g-C_3_N_4_@PdS-3, measuring 1.23 and 0.87 eV, respectively. Utilizing the formula E_VB, NHE_ = φ + E_VB, XPS_ − 4.44, where φ signifies the work function of the XPS instrument (φ = 4.258), and E_VB, NHE_ represents the valence band position relative to the normal hydrogen electrode (NHE), the calculated E_VB, NHE_ are 1.05 and 0.688 eV for g-C_3_N_4_ and g-C_3_N_4_@PdS-3, respectively. These results collectively confirm that the conduction band of g-C_3_N_4_@PdS heterojunction exists in a more negative position, which inherently favors a hydrogen evolution reaction.

**Figure 6:**
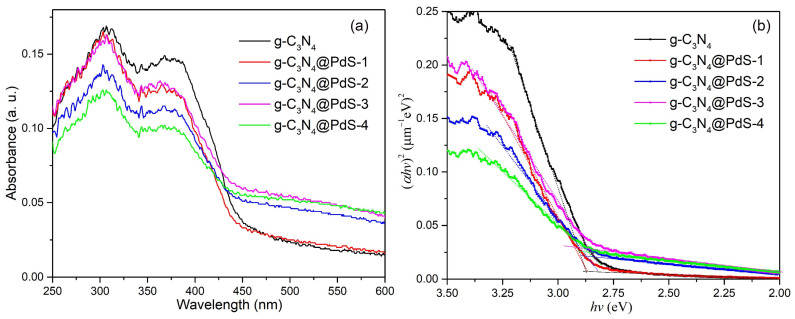
(**a**) UV-vis spectra, and (**b**) the corresponding Taus plots of the g-C_3_N_4_ and g-C_3_N_4_@PdS nanocomposites.

To determine the optical band gap of the g-C_3_N_4_ and g-C_3_N_4_@PdS nanocomposites, we conducted measurements using the ultraviolet-visible diffuse scattering spectrum, displayed in Figure 6a. This spectrum exhibits a distinct absorption edge, demonstrating a redshift in the absorption edge as the load of PdS nanoparticles increases. This indicates an enhanced light absorption capability in g-C_3_N_4_@PdS nanocomposites, visibly reflected in their color transformation. As depicted in Figure S1 of the supporting material, the transition from the whitish-yellow hue of g-C_3_N_4_ to brown reinforces the heightened light absorption capacity of the nanocomposite material. This augmented light absorption implies a more efficient utilization of light in the generation of photogenerated electrons and holes during the photocatalytic H_2_ production process. The band gap of the both g-C_3_N_4_ and g-C_3_N_4_@PdS nanocomposites was determined using the Tauc equation [54]: *αhv* = A(*hv* − E_g_)^1/2^, where E_g_ represents the optical bandgap, *α* signifies the absorption coefficient, *h* and *v* denote Planck’s constant and incident light frequency, respectively, while A represents a constant. Figure 6b illustrates the correlation between *hv* and (*αhv*)^2^. According to the Tauc equation, the optical band gap is identified at the intersection of the linear segment of the curve with the base line. For g-C_3_N_4_, g-C_3_N_4_@PdS-1, g-C_3_N_4_@PdS-2, g-C_3_N_4_@PdS-3, and g-C_3_N_4_@PdS-4, the optical band gaps measure 2.874, 2.869, 2.866, 2.861, and 2.854 eV, respectively.

**Figure 7:**
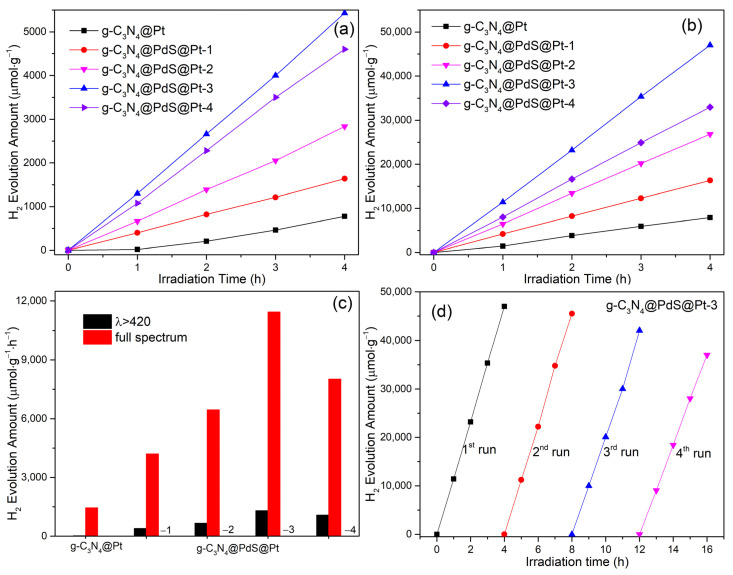
The relationship of the amount of photocatalytic H_2_ evolution with time under the irradiation of a xenon lamp light source: (**a**) with long pass filter (*λ* > 420 nm) and (**b**) without filter; (**c**) the photocatalytic H_2_ evolution rates of g-C_3_N_4_@Pt and g-C_3_N_4_@PdS@Pt photocatalysts with various loading amounts of PdS under visible light and full spectrum irradiation; (**d**) cycling stability test of the photocatalytic H_2_ evolution for g-C_3_N_4_@PdS@Pt-3 under full spectrum irradiation.

The effectiveness of g-C_3_N_4_ nanocomposites in photocatalytic water splitting was evaluated under varying PdS nanoparticle loads at a temperature of 5 °C in the presence of 20 vol % lactic acid. Lactic acid was utilized as a sacrificial agent to capture the holes in the process. Before evaluating the performance of photocatalytic H_2_ production, a co-catalyst, Pt, was applied to the catalyst via photodeposition, where chloroplatinic acid was used as the Pt source. Figure 7a,b display the corresponding H_2_ generation rate of the prepared photocatalyst under visible light (using xenon lamp irradiation with a long pass filter, *λ* > 420 nm) and full spectrum (xenon lamp irradiation) over time. The graphs illustrate a linear increase in H_2_ production for all photocatalysts with extended exposure to both light sources. Particularly under visible light, it is evident that the g-C_3_N_4_@Pt catalyst exhibits the lowest H_2_ production compared to g-C_3_N_4_@PdS@Pt nanocomposites. This lower H_2_ output is attributed to the rapid recombination of photogenerated carriers in g-C_3_N_4_. Remarkably, the g-C_3_N_4_@PdS@Pt-3 nanocomposites exhibit exceptional properties, generating up to 1289 μmol·g^−1^·h^−1^, a staggering 60-times increase compared to g-C_3_N_4_@Pt (20.9 μmol·g^−1^·h^−1^). Upon removing the filter, the H_2_ production efficiency notably surges under full spectrum irradiation, as depicted in Figure 7b. The H_2_ production rate of g-C_3_N_4_@PdS@Pt-3 reaches an impressive 11438 μmol·g^−1^·h^−1^, which is eight times higher than the H_2_ production efficiency of g-C_3_N_4_@Pt (1452 μmol·g^−1^·h^−1^) under identical conditions. This showcases a ninefold improvement over visible light irradiation. Figure 7c clearly depicts the trend in H_2_ production efficiency under the two light sources. To ascertain the chemical stability of the photocatalyst, the stability of g-C_3_N_4_@PdS@Pt nanocomposites was tested over four cycles under full spectrum irradiation (Figure 7d). The results indicate that after two cycle tests the photocatalytic H_2_ production performance remains largely unchanged. However, during the third cycle, there is a slight decrease in performance due to the consumption of lactic acid in the reaction solution. Table 1 outlines the comparison between the H_2_ production efficiency achieved by combining g-C_3_N_4_ with various catalysts and the optimal efficiency observed in this study. Evidently, the introduction of the PdS and Pt co-catalysts significantly enhances the performance of photocatalytic H_2_ production.

**Figure 8:**
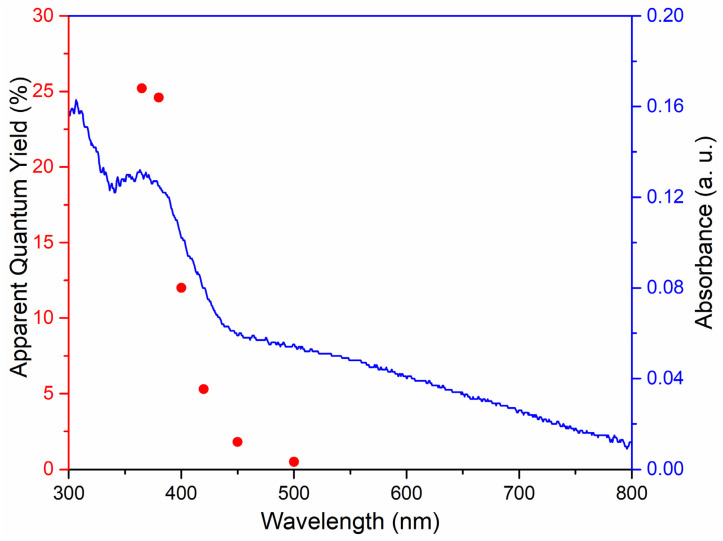
The UV-vis absorption spectrum and AQE data of the g-C_3_N_4_@PdS@Pt-3 catalyst.

The apparent quantum efficiency (AQE) was calculated using the following equation:

(1) $$AQYs(\%)=\frac{2\times N_{H_{2}}}{N_{p}}\times 100\%=\frac{2\times N_{H_{2}}}{\frac{I\times A\times \lambda }{h\times c}}\times 100\%$$

where *N_p_*, *I*, *A*, *h*, *c*, and *λ* represent the number of incident photons, the illumination intensity, the irradiation area of the incident light, Planck’s constant, the speed of light, and the wavelength of the incident light, respectively. Here, the monochromatic light was achieved by implementing a band-pass filter in the xenon light source outlet. According to Formula 1, the AQY of g-C_3_N_4_@PdS@Pt-3 catalyst at 365, 380, 400, 420, 450, and 500 nm were calculated and are depicted in Figure 8. As illustrated in Figure 8, the quantities of H_2_ produced at different wavelengths correlate with the light absorption, showcasing a decrease in AQE as the wavelengths increase. Notably, the highest AQE, recorded at 365 nm, stands at an impressive 25.2%.

**Figure 9:**
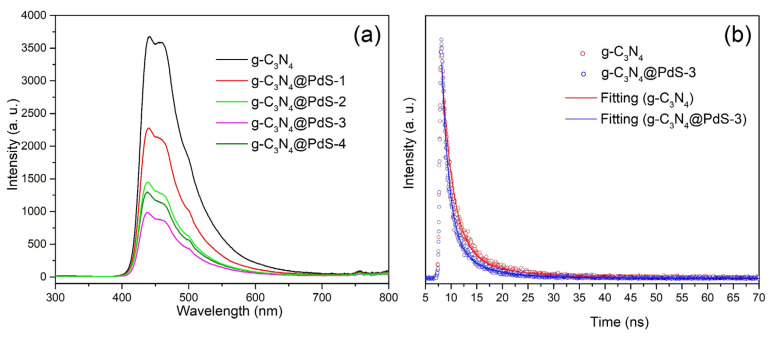
(**a**) PL spectra of g-C_3_N_4_, g-C_3_N_4_@PdS-1, g-C_3_N_4_@PdS-2, g-C_3_N_4_@PdS-3, and g-C_3_N_4_@PdS-4 nanocomposites; (**b**) TRPL decay curves of g-C_3_N_4_ and g-C_3_N_4_@PdS-3 nanocomposites.

The separation characteristics of carriers within the samples are investigated using PL and TRPL measurements. Figure 9a illustrates the PL spectrum at a 250 nm excitation wavelength, highlighting a primary blue luminescence peak at ≈480 nm, which originates from the transition between lone pair states in the valence band and the π* antibonding states in the conduction band [61], indicating charge recombination. In the figure, the bare g-C_3_N_4_ display the most pronounced PL peak, while the PL intensities noticeably decrease upon the integration of PdS. Notably, the g-C_3_N_4_@PdS-3 hybrid exhibits the lowest PL intensity among all the nanocomposites, consistent with the comparison of photocatalytic activities. This analysis indicates that the g-C_3_N_4_@PdS nanocomposites effectively mitigate the charge recombination in g-C_3_N_4_. Furthermore, the TRPL experiments were conducted on both bare g-C_3_N_4_ and g-C_3_N_4_@PdS-3 samples to delve deeper into the charge transmission process, as depicted in Figure 9b. The TRPL curves were fitted using a multiexponential function, and the resulting parameters are summarized in Table 2. Decay lifetimes were calculated according to Equation (2):

(2) $$\tau =\frac{A_{1}\cdot \tau_{1}^{2}+A_{2}\cdot \tau_{2}^{2}}{A_{1}\cdot \tau_{1}+A_{2}\cdot \tau_{2}}$$

where, *τ*_1_, *τ*_2_ represent the short carrier lifetime attributed to quasi-free excitons and the long component due to localized exciton recombination, respectively. *A*_1_ and *A*_2_ correspond to the percentages of the short and long component in the total lifetime. Significantly, the bare g-C_3_N_4_ (5.7917) exhibits a longer average decay lifetime compared to g-C_3_N_4_@PdS-3 (7.8609 ns). The brief fluorescence lifetime hints at the possibility of extra non-radiative attenuation pathways being activated within the g-C_3_N_4_@PdS sample. These pathways could effectively impede the recombination of photogenerated carriers. The outcomes mentioned above demonstrate that the heterojunction created by embedding PdS nanoparticles onto g-C_3_N_4_ nanosheets actively facilitates the parting of electron-hole pairs, consequently enhancing the photocatalytic efficiency.

**Figure 10:**
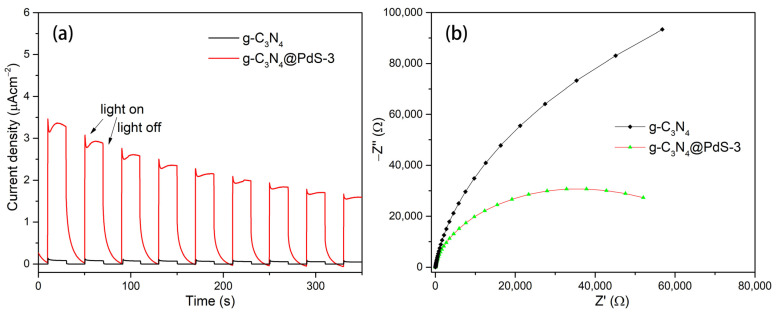
(**a**) Transient photocurrent responses spectra under Xe lamp, (**b**) EIS Nyquist spectra of g-C_3_N_4_ and g-C_3_N_4_@PdS-3 nanocomposites.

**Figure 11:**
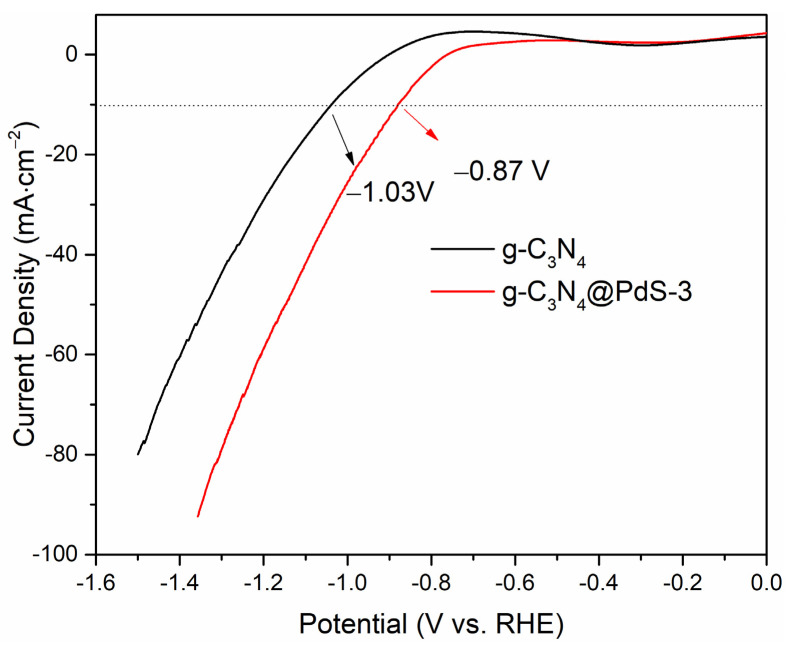
LSV curves of the g-C_3_N_4_ and g-C_3_N_4_@PdS nanocomposites.

Furthermore, this study delved into the interfacial charge transfer and separation capacities of both pure g-C_3_N_4_ and g-C_3_N_4_@PdS nanocomposites through analyses using transient photocurrent and electrochemical impedance spectroscopy. As depicted in Figure 10a, the g-C_3_N_4_@PdS-3 nanocomposites demonstrated a notably heightened photocurrent response compared to the pristine g-C_3_N_4_, indicating significantly enhanced charge separation capabilities. Additionally, electrochemical impedance spectroscopy (EIS) can also be used to assess electron mobility at the electrode interface, which usually reflects the charge transfer ability of the photocatalyst. The ESI Nyquist diagram in Figure 10b vividly illustrates that the arc radius of the g-C_3_N_4_@PdS-3 nanocomposites is markedly smaller than that of the pure g-C_3_N_4_, signifying swifter charge transfer kinetics and lower charge transfer resistance in the former. In addition, Figure 11 provides a comparison of the overpotential of g-C_3_N_4_ and g-C_3_N_4_@PdS through linear scanning voltammetry (LSV) measurements. At the reference current density of 10 mAcm^−2^, g-C_3_N_4_@PdS-3 exhibits a lower overpotential (−0.87V) compared to g-C_3_N_4_(−1.03V), suggesting that the presence of PdS loaded onto g-C_3_N_4_ is more favorable for facilitating H_2_ production than g-C_3_N_4_ alone.

**Figure 12:**
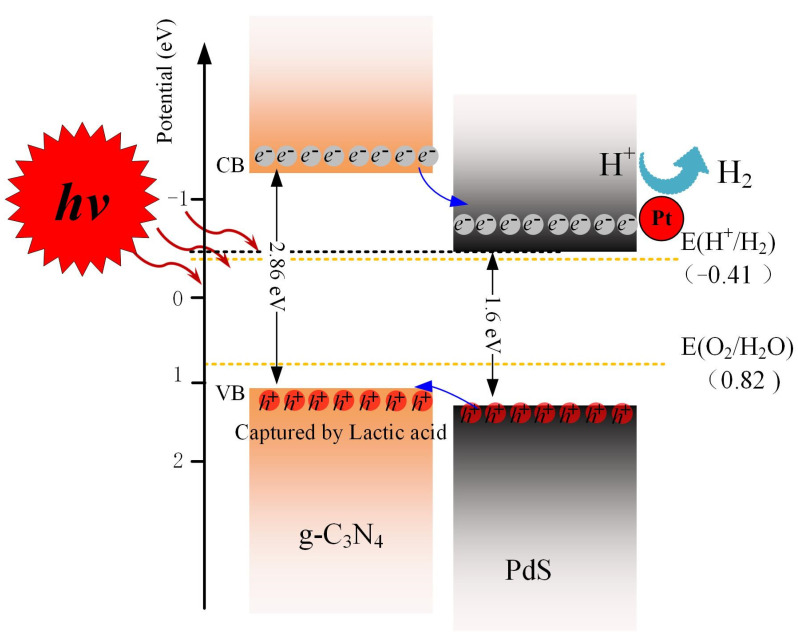
Proposed photocatalytic mechanism for the g-C_3_N_4_@PdS@Pt nanocomposites.

It has been reported that PdS is an n-type semiconductor with a band gap of 1.6 eV. Its valence and conduction band positions are situated at 1.1 eV and −0.5 eV, respectively [62,63]. Drawing upon the preceding analyses, Figure 12 outlines a reasonable charge transfer behavior and proposes a mechanism for photocatalytic hydrogen production reaction in g-C_3_N_4_@PdS@Pt nanocomposites. Initially, the attachment of PdS uniformly onto g-C_3_N_3_ nanosheets widens the light absorption spectrum of nanocomposites. Therefore, the inclusion of PdS augments the production of photogenerated charge carriers. Additionally, the uniform distribution of PdS enhances the availability of active sites. Upon exposure to light, incident light energizes valence band electrons into the conduction band while generating holes in the valence band. Subsequently, electrons within the conduction band of g-C_3_N_4_ swiftly migrate to the conduction band of PdS, where they are captured by Pt, catalyzing a reduction reaction upon interaction with absorbed hydrated protons at the Pt site, thereby liberating hydrogen. Simultaneously, the holes present in the valence band are continually consumed with the electron donor, lactic acid in the solution. Hence, the existence of PdS enhances the quantity of photoinduced carriers and effectively facilitates their separation.

## 3. Experimental

### 3.1. Materials

Melamine (99%), sodium chloropalladate (Na_2_PdCl_4_, 99.99%), sodium sulfide (Na_2_S, 99%), ethanolamine (99%), lactic acid (20%), chloroplatinic acid hexahydrate (AR, Pt > 37.5%), and sodium sulfate (AR, 99%) were purchased from Aladdin Reagent Co., Ltd. (Shanghai, China) and employed directly without additional purification. Deionized water with 18 MΩ cm was used in our experiment.

### 3.2. Synthesis of g-C_3_N_4_@PdS Nanocomposites

**Scheme 1:**
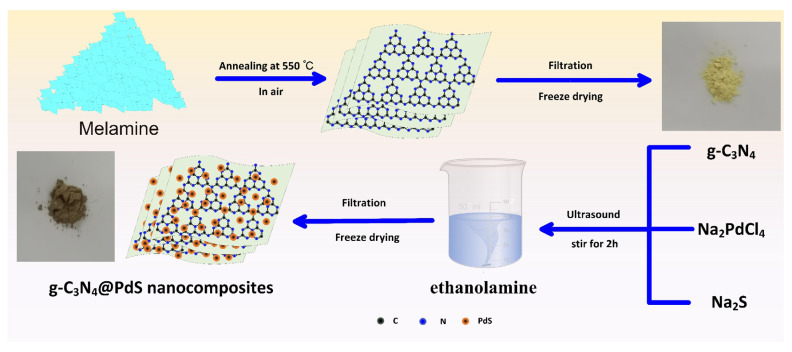
Schematic depiction of the synthesis process for g-C_3_N_4_@PdS nanocomposites.

The synthesis process of g-C_3_N_4_@PdS nanocomposites is presented in Scheme 1. Briefly, g-C_3_N_4_ nanosheets were synthesized via a double pyrolysis process of melamine, as detailed in our previously published literature [20]. Its color is milky yellow, as shown in Scheme 1. Subsequently, 400 mg of g-C_3_N_4_ underwent ultrasonic dispersion in 50 mL of ethanolamine for 2 h. Concurrently, a solution containing 0.01 mmol of Na_2_PdCl_4_ dissolved in 10 mL of deionized water was vigorously stirred to achieve uniformity. This solution was gradually added drop by drop to the ultrasonically dispersed g-C_3_N_4_ solution. Following 2 h of stirring, 0.6 × 10^−3^ M Na_2_S was incrementally introduced into the solution. The resultant mixture was thoroughly stirred for 12 h, washed successively with ethanol and deionized water, and then freeze-dried, resulting in the sample denoted as g-C_3_N_4_@PdS-1. To find the optimal PdS load, a series of nanocomposites were synthesized. As the quantity of the Pd source was incrementally altered to 0.02, 0.03, and 0.04 mmol (with the corresponding S source being adjusted to 1.2 × 10^−3^ M, 2.4 × 10^−3^ M, and 3.6 × 10^−3^ M, respectively), the synthesized samples were sequentially designated as g-C_3_N_4_@PdS-2, g-C_3_N_4_@PdS-3, and g-C_3_N_4_@PdS-4. Scheme 1 depicts the color of g-C_3_N_4_@PdS nanocomposites, exhibiting a light brown hue. This coloration signifies an enhancement in the nanocomposites’ capability to absorb visible light.

### 3.3. Characterization

XRD analysis was performed using a Bruker D8 Advance instrument (Bruker, Saarbrucken, Germany) to assess the evolution of the crystal structure following the incorporation of PdS. Morphologies and microstructural investigations were conducted using Talos F200X G2 TEM JEOL (Thermo Scientific, Waltham, MA, USA, FEI company, Hillsboro, OR, USA), and HRTEM (Thermo Scientific, Waltham, MA, USA, FEI company, Hillsboro, OR, USA) operated at an accelerated voltage of 200 KV. Additionally, super-X model EDS accompanied TEM analysis to investigate the element distribution. For TEM analysis, a 1 mg catalyst dispersed in ethanol underwent ultrasonication for 10 min, was deposited onto a copper grid, naturally dried, and then examined. The functional group characteristics of the synthesized materials were analyzed via FTIR, Thermo Scientific Nicolet iS20 (Thermo Scientific, Waltham, MA, USA). Furthermore, changes in the valence state and band structure of the elements in the nanocomposite were explored using XPS via a PHI 5000 Versaprobe Ⅲ (Spectra Research Corporation, Mississauga, ON, USA) spectroscopy instrument, utilizing monochromatic Al Kα radiation. Ultraviolet-visible diffuse reflectance spectra were acquired using a Hitachi UH4150 (Hitachi High-Tech Corporation, Tokyo, Japan) equipped with an integrating sphere. Steady-state PL was investigated using the Hitachi F7000 spectrofluorometer (Hitachi High-Tech Corporation, Tokyo, Japan), employing an excitation wavelength of 250 nm. Evaluation of photocurrent performance, EIS and LSV were conducted using a three-electrode electrochemical workstation (CHI660E, Chen Hua, Shanghai, China). The reference electrode used was Ag/AgCl, while the counter electrode utilized a Pt plate. To prepare the working electrode, 5 mg of the catalyst was ultrasonically dispersed in 1 mL of ethanol along with 20 μL of nafion solution, forming a uniform solution. This catalyst-containing solution was subsequently deposited onto the FTO glass and dried to form the working electrode. The electrolyte employed was a 0.5 M aqueous solution of Na_2_SO_4_. A bias voltage of 0.3 V was added to the photocurrent test. The potential of the electrode of EIS test voltage was 0.24 V.

### 3.4. Evaluation of Photocatalytic H_2_ Production Activity

The procedure for measuring photocatalytic H_2_ production involved using a reaction flask filled with 30 mg of the photocatalyst alongside a 20% lactic acid aqueous solution (10 mL) serving as a sacrificial agent. This was combined with 100 mL of deionized water and a 3% wt Pt, employed as a co-catalyst, with chloroplatinic acid as the Pt source. The mixture was subjected to ultrasonic dispersion for 30 min to ensure the formation of a uniformly dispersed suspension. This suspension was then transferred into a quartz reactor connected to an on-line trace gas analysis system (Labsolar-6A, Beijing Perfectlight, Beijing, China). A constant temperature water-cooling system was used to maintain the reaction solution at 5 °C. To guarantee the complete elimination of air, the system and the reactor were evacuated several times. After the vacuum extraction, the reactor underwent a 30 min exposure to a 300 W xenon arc light source to facilitate the reduction of Pt. Subsequently, Pt was loaded onto the g-C_3_N_4_ and g-C_3_N_4_@PdS nanocomposites. The resulting photodeposited nanocomposites were sequentially labeled as g-C_3_N_4_@Pt, g-C_3_N_4_@PdS@Pt-1, g-C_3_N_4_@PdS@Pt-2, g-C_3_N_4_@PdS@Pt-3, and g-C_3_N_4_@PdS@Pt-4. After light deposition, the system was vacuumed again in preparation for photocatalytic H_2_ production experiments. The photocatalytic H_2_ production experiments commenced with the reactor being irradiated under visible light (using xenon lamp irradiation with a long pass cut-off filter, *λ* > 420 nm) and full spectrum (xenon lamp irradiation). Following irradiation, the concentration of photocatalytically produced H_2_ was assessed using an online gas chromatograph (Fuli instruments, Zhejiang, China, GC9720PLUS) equipped with a thermal conductive detector.

## 4. Conclusions

In this paper, a g-C_3_N_4_@PdS nanocomposite with varying concentration of PdS was prepared via a straightforward method. Comprehensive investigations into the microstructure, morphology, band structure, element distribution, and optical properties of these catalysts were conducted. XPS analysis unambiguously confirmed the successful loading of PdS onto the g-C_3_N_4_ layer, while TEM imaging revealed the uniform distribution of PdS nanoparticles on the g-C_3_N_4_ layer. The crystal structure of the resultant g-C_3_N_4_@PdS nanocomposite remained largely unchanged, attributed to the low PdS content. The study extensively evaluated the photocatalytic performance of these nanocomposites under both visible light and full spectrum irradiation. Encouragingly, the g-C_3_N_4_@PdS composites exhibited significantly enhanced photocatalytic hydrogen production. Notably, the hydrogen production rate of g-C_3_N_4_@PdS@Pt-3 nanocomposites surpassed that of g-C_3_N_4_@Pt by 60-times under visible light and 8-times under full spectrum irradiation. Characterization through various methods including photocurrent response curve, EIS, LVS, PL, and TRPL decay curves unveiled that the structure of the composite facilitated an accelerated transfer of photogenerated carriers, thereby augmenting the photocatalytic hydrogen production rate. The incorporation of PdS enhances light absorption and enhances the efficiency of carrier transfer, thereby contributing to the improved performance of the g-C_3_N_4_@PdS@Pt nanocomposite.

## Figures and Tables

**Table 1:** Comparative survey of photocatalytic H_2_ evolution performance: g-C_3_N_4_@PdS@Pt-3 versus other reported photocatalysts.

Catalyst	Synthesis Method	Dosage (mg)	Type of Light Source	Sacrificial Reagent	H_2_ Evolution Rate (μmol·g^−1^·h^−1^)	Refs.
Au@g-C_3_N_4_	Solution–precipitation method	20	365 nm wavelength light excitation	10% Triethanolamine	530	[55]
Au@g-C_3_N_4_	Facile deposition–precipitation method	20	125W medium pressure visible-light Hg Lamp	10% Triethanolamine	177.4	[56]
Pt@g-C_3_N_4_	Photodeposition of Pt	8	300 W xenon Lamp	8 mL of TEOA solution	4210.8	[57]
Pd-NVs-C_3_N_4_	Photoreduction method	100	300 W xenon Lamp	20 vol% methanol	287.9	[58]
graphdiyne/g-C_3_N_4_	Calcination method	50	300 W xenon Lamp (*λ* > 420 nm)	15% Triethanolamine	39.6	[59]
MoS_2_@g-C_3_N_4_	Probe sonication-assisted liquid exfoliation method	50	300 W xenon Lamp (*λ* > 420 nm)	0.1M Triethanolamine	1155	[60]
g-C_3_N_4_@PdS@Pt-3	Precipitation method	30	300 W Xe arc Lamp (*λ* > 420 nm)	20% lactic acid aqueous solution	1289	This work
			300 W Xe arc Lamp		11,438	

**Table 2:** Dynamic analysis of emission decay for g-C_3_N_4_, andg-C_3_N_4_@PdS-3 nanocomposites.

Sample	*τ*_1_ (ns)	*A*_1_ (%)	*τ*_2_ (ns)	*A*_2_ (%)	*τ* (ns)
g-C_3_N_4_	2.1714	57.51	9.6023	42.49	7.8609
g-C_3_N_4_@PdS-3	1.5004	52.84	6.8455	47.16	5.7917

## Data Availability

Data are contained within the article and Supplementary Materials.

## Supplementary Materials

The following supporting information can be downloaded at: www.mdpi.com/xxx/s1.
