# Supplementary material for: Achieving Highly Efficient Photocatalytic Hydrogen Evolution through the Construction of g-C3N4@PdS@Pt Nanocomposites
Source: Molecules. 2024 Jan 19;29(2):0. doi: 10.3390/molecules29020493 (PMC11154439; doi:10.3390/molecules29020493)
Supplement: Supplementary file 1 [file molecules-29-00493-s001.zip › molecules-2820114-supplementary.pdf]

Supporting materials for

Achieving Highly Efficient Photocatalytic Hydrogen Evolution through the Construction of g-C_3N_4@PdS@Pt Nanocomposites

Ligang Ma [1], Chao Lin [1], Wenjun Jiang [1], Shun Yan [1], Huilin Jiang [1], Xiang Song [1], Xiaoqian Ai [2,*], Xiaoxiao Cao [2] and Yihuan Ding [1]

[1] School of Electronic Engineering, Nanjing Xiaozhuang University, Nanjing 211171, China
[2] School of Physics and Information Engineering, Jiangsu Province Engineering Research Center of Basic Education Big Data Application, Jiangsu Second Normal University, Nanjing 210013, China
* Correspondence: aixiaoqian186@jssnu.edu.cn

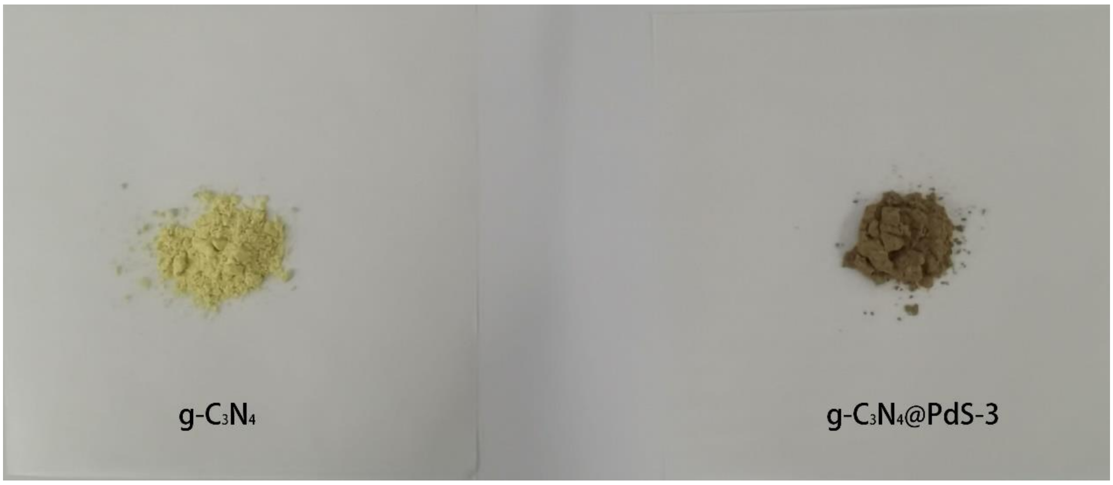

Figure S1. Real photos of g-C_3N_4 and g-C_3N_4@PdS-3 nanocomposite.
